# Examining Pregnancy Related Socio-Cultural Factors Among Adolescent Girls in the Komenda-Edina-Eguafo-Abrem Municipality in the Central Region of Ghana: A Case-Control Study

**DOI:** 10.3389/fpubh.2019.00093

**Published:** 2019-04-24

**Authors:** Bright Opoku Ahinkorah, John Elvis Hagan Jr., Abdul-Aziz Seidu, Joseph Kwame Mintah, Francis Sambah, Thomas Schack, Thomas Hormenu

**Affiliations:** ^1^Australian Centre for Public and Population Health Research, University of Technology Sydney, Ultimo, NSW, Australia; ^2^Department of Health, Physical Education, and Recreation, University of Cape Coast, Cape Coast, Ghana; ^3^Neurocognition and Action-Biomechanics-Research Group, Faculty of Psychology and Sport Sciences, Bielefeld University, Bielefeld, Germany; ^4^Department of Population and Health, University of Cape Coast, Cape Coast, Ghana; ^5^National Institutes of Health, Section of Ethnicity and Health, DEOB, NIDDK, Bethesda, MD, United States

**Keywords:** socio-cultural, pregnancy, adolescents, Central region, Ghana

## Abstract

**Background and Purpose:** Given that Ghana continues to record higher rates of adolescent pregnancy among rural dwellers, notably, those living in the Brong Ahafo, Central and Volta regions, it is surprising that scholarly information related to socio-cultural determinants of this sequel in these mentioned areas of the country is limited. This study sought to examine the socio-cultural factors associated with pregnancy among adolescent girls in Komenda-Edina-Eguafo-Abrem (KEEA) Municipality in the Central Region of Ghana.

**Methods and results:** Using a matched case-control design with a 1:1 ratio, a facility-based sampling approach was used to select 400 adolescent females aged between 15 and 19 years. Chi-square analyses on the strictness level of rules and regulations in family [χ^2^_(1)_ = 62.010, *p* < 0.05], freedom within the family to discuss issues related to sexuality [χ^2^_(1)_ = 9.600, *p* < 0.05], religious support of sex before marriage [χ^2^_(1)_ = 4.312, *p* < 0.05], peer influence to engage in sexual intercourse [χ^2^_(1)_ = 7.793, *p* < 0.05], reaction of parents toward pregnancy [χ^2^_(1)_ = 70.064, *p* < 0.05], and reaction of siblings toward pregnancy [χ^2^_(1)_ = 66.702, *p* < 0.05] were significantly related to adolescents' pregnancy status. Additionally, binary logistic regression analysis also showed that non-pregnant adolescents were seven times more likely to belong to families with strict rules and regulations compared to pregnant adolescents [OR = 0.14, 95% CI = (0.07–0.25), *p* ≤ 0.01]. Non-pregnant adolescents were 3 times more likely to have freedom within the family to discuss issues related to sexuality compared to pregnant adolescents [OR = 0.33, 95% CI = (0.18–0.59), *p* ≤ 0.01]. However, pregnant adolescents were 3 times more likely to be influenced by their peers to engage in sexual intercourse [OR = 2.63, 95% CI = (1.46–4.74), *p* ≤ 0.001] and 3 times more likely to have parents with positive reaction toward adolescent pregnancy compared to non-pregnant adolescents [OR = 2.97, 95% CI = (1.15–7.70), *p* ≤ 0.05]. Additionally, these pregnant adolescents were eight times more likely to have siblings with positive reaction toward adolescent pregnancy [OR = 7.74, 95% CI = (2.59–21.4), *p* ≤ 0.001] compared to their non-pregnant counterparts.

**Conclusion:** Adolescent pregnancy heightens the risk of negating birth outcomes that is independent of recognized confounding variables. Therefore, adolescent girls in the KEEA Municipality are likely to experience continuous exposure to the risk of pregnancy with the existence of negative socio-cultural norms. Specific strategies need to involve multifaceted interventions that include education, competency-based skill training and support for young people, especially pregnant adolescents. Further research ought to determine which other factors would help us better understand circumstances that may lead to adolescent pregnancies in other areas of the region and perhaps among other subgroups.

## Introduction

Adolescent pregnancy appears today as a complex tapestry where different dimensions interact. Adolescence is also defined by UNICEF (1), as a transition from childhood to adulthood often characterized by physical, psychological, and social changes. It is generally classified into two: early adolescence (10–14 years) and late adolescence (15–19 years). A major test at this stage of growth is that fresh feelings develop, friends assume greater importance, and interest builds in the opposite sex (2, 3). World Health Organization (4) defines adolescent pregnancy as pregnancy occurring in a girl aged 10–19 years. This pregnancy can occur among early adolescents (10–14 years) and late adolescents (15–19 years). According to Erikson's theory of psychosocial development, adolescents from 12 onwards are saddled with a psychosocial crisis called identity vs. role confusion that may be experienced in adolescent pregnancy (3, 5).

Globally, about 16 million adolescents aged between 15 and 19 years give birth each year, accounting for 11% of all births worldwide (6, 7). Approximately 95% of adolescent pregnancies occur in low middle income countries (LMIC). Significant geographical variations exist; for instance, births to adolescents as a percentage of all births range from approximately 2% in China, to 18% in Latin America and the Caribbean, to more than 50% in Sub-Saharan Africa (8). Pregnancy cases among young mothers have become a significant burden, with statistics from LMICs suggesting that nearly 10% (i.e., 36.4 million) of girls become mothers by age 16. The highest proportions in the world were noted in Sub-Saharan Africa, South Central and Southeast Asia (8, 9). By 2013, Sub-Saharan Africa had the highest prevalence of adolescent pregnancy in the world in 2013 (9). Births to teenage mothers accounted for more than half of all the births in this region: an estimated 101 births per 1,000 women aged 15–19 (9). Most countries with teenage pregnancy levels more than 30% occur in sub-Saharan Africa (10), birth rates ranged from 150 or more to <50 births per 1,000 women of ages 15–19 in sub-Saharan Africa, with Central Africa experiencing the highest levels and Southern Africa having the lowest level (11).

The current situation of adolescent pregnancy in Ghana is not different from other developing societies. Adolescent pregnancy is endemic in Ghana. The Ghana Demographic and Health Survey 2014 report indicates that the rate of pregnancy among adolescents aged 15–19 was high, with rural dwellers and those living in the Brong Ahafo, Central and Volta regions recording the highest rates (12). This report reiterated that the number of adolescents who were pregnant with one child in the Central Region was 7%. Within the Central Region, the Komenda-Edina-Eguafo-Abrem (KEEA) Municipality is one of the districts that have high prevalence of adolescent pregnancy. In 2016, out of the total pregnancies in the Municipality, 17.5% were adolescents (13). The past decade has seen no significant change in the rates reported between 2003 (14%) and 2008 (13%) (14, 15). For example, about one in ten adolescents aged between 15 and 19 years had begun childbearing in urban areas in Ghana whilst about twice the number was the problem in the rural communities in 2009 (15). These trends show that Ghana continues to record higher rates of adolescent pregnancy.

Studies have identified sexual health knowledge, attitude, behavior, and health service accessibility, and acceptability as broad risk factors for pregnancy among adolescents (16, 17). Besides, other researchers have identified diverse socio-cultural factors such as family structure, peer group influence, cultural permissiveness, family instability, early age of marriage, lack of knowledge of sexuality, lack of knowledge, and/or ineffective use of contraceptives to influence pregnancy among adolescents (18, 19). For example, East et al. (18) investigated the association between adolescent pregnancy and family history of teenage births. After controlling for socioeconomic and mothers' parenting characteristics, findings showed that adolescents whose mothers and sisters once had teenage births were at a higher risk of getting pregnant. Additionally, four maternal parenting measures (being single, lax parenting, approval of teenage sex and parenting, and low level of education) were significantly associated with study participants' increased risk of teenage pregnancy. Similarly, Osaikhuwuomwan and Osemwenkha (19) found in Nigeria that peer group pressure to have sex had the highest rating (71.8%) on participants perceptions on the causes of adolescent pregnancy. Other items rated were ignorance on basics of sexuality and pregnancy (60.1%) as well as reported poor uptake of contraception (69.3%) by adolescents. Other studies found varied socio-cultural risk factors associated with high pregnancy rate among adolescents. The level of strictness of rules and regulations in the family, level of freedom within the family to discuss problems regarding love affairs; level of freedom within the family to discuss issues related to sexuality, parental attitudes toward sex, ineffective parenting, and poverty have been cited (20–22).

The negative health, psychosocial, and economic impact connected with adolescent pregnancy is worrisome and should not be underestimated. Adolescent pregnancy brings sudden change in the lives of young girls and in most cases negative consequences such as dropping out of school and interrupted education. Psychologically, these young girls experience higher levels of stress, despair, depression, feelings of helplessness, low self-esteem, a sense of personal failure, and suicide attempts than perhaps their older counterparts (19). Further, pregnant adolescents face increased stereotyping and stigma as young mothers. These negative behaviors can increase the risk of adolescent mothers' not accessing health services and becoming more socially isolated (23). Besides medical risks (e.g., dysfunctional labor, pregnancy-related infections, postpartum hemorrhage, premature rupture of membrane, and higher rates of premature and/or low birth weight babies), the economic implications for the adolescent mother and her child are of great consequence; poverty; unemployment and poor literacy among others (19, 24).

Therefore, an empirical enquiry on socio-cultural determinants associated with pregnancy in adolescent girls in one of the endemic areas (KEEA Municipality) of the Central Region of Ghana would be worthwhile. This research investigation would provide some baseline information to strengthen the knowledge base and help policy makers set innovative community-wide approaches to reduce adolescent pregnancies through evidence-based prevention programs within this geographical area.

## Materials and Methods

### Study Design

A matched case-control study design with a 1:1 ratio was used for the study. A case was defined as any female adolescent (aged 15–19 years) who was pregnant and accessed healthcare in any of the health facilities in the KEEA Municipality in this study. Controls were defined as girls within adolescence (at the time of data collection) who have never been pregnant. We justify the use of this design because it enables researchers to identify risk factors associated with health behaviors such as adolescent pregnancy by explicitly focusing on cases (adolescent group known to have a pregnancy outcome) and the controls (adolescent group known to be free of pregnancy outcome). Then, we look back in time to learn which subjects in each group had the exposure comparable to studied variables. Ethical approval for this study was sought from the Research and Ethics Committee of University of Cape Coast, Ghana (UCCIRB/CES/2016/04) and the Ghana Health Service Ethics Review Committee (GHS-ERC: 13/12/2016). Permission to carry out the study was also obtained from the Central Regional Health Directorate, the KEEA Municipality Health Directorate and the health facilities involved in the study.

### Study Setting

The study setting was the KEEA Municipality. According to the 2010 Population and Housing Census, the population of KEEA Municipality was 144,705. The population represented 6.6% of the region's total population. Males constitute 48.2% whereas females represent 51.8%. Sixty four percent (64%) of the population is rural. The population of the district is youthful because 40.2% of the population is below 15 years, depicting a broad base population pyramid which tapers off with a small number of elderly persons (8.6%). This Municipality has a General Fertility Rate of 105.0 births per 1,000 females aged 15–49 years and a Total Fertility Rate of 3.6. The Crude Birth Rate (CBR) of the Municipality is 24.6 per 1,000 population (25).

### Population and Sampling

The population for the study was made up of female adolescents (15–19) years in the KEEA Municipality. A total population of 7,667 females (10–19 years), comprising both pregnant and non-pregnant adolescents was used for the study (25). A facility based sampling approach was used to select 400 female adolescents made up of 200 pregnant and 200 non-pregnant adolescents. This sample size was determined using Krejcie and Morgan (26) table of determining sample sizes. With this sampling approach, pregnant adolescents accessing antenatal services from five health facilities in the municipality were initially purposively and conveniently selected. The health facilities were Kissi health center, Komenda health center, Elmina urban health center, Agona health center and Ankaful General Hospital. For each pregnant adolescent selected, a non-pregnant adolescent within the same age group was conveniently identified from the five health facilities. The number of pregnant adolescents and non-pregnant adolescents sampled constituted the number of cases and controls for the study. The choice of the facility-based sampling was because it was difficult to reach potential participants using other sampling techniques.

### Instrumentation

A literature review was the source from which questionnaire items were generated. The questionnaire consisted of preliminary information on participants' demographics (e.g., religion, mother and father's educational status, employment status). Other items on the instrument were socio-cultural factors related to pregnancy within which respondents were to answer with a binary response (e.g., Yes or No). Some specific examples of items on the instrument were “Freedom within the family to discuss issues related to sexuality; Religious support of sex before marriage.” Face, construct and content validity were ensured by presenting the pre-test version of the questionnaire to three experienced professors in adolescent health education and promotion for their review and input to ensure that the individual item construction and whole content of the questionnaire mirrored the exact rational of the study (27). The questionnaire was then pre-tested using 50 adolescents in the Cape Coast Metropolis. Using the Kuder-Richardson formula 21 (KR-21), the instrument yielded a reliability coefficient of 0.71, a figure that is deemed acceptable (28).

### Procedure

An initial arrangement was made with the medical directors of the five (5) designated health centers to collect data from potential females. Two of the researchers and two trained assistants visited these health posts and collected data consistently for 12 weeks. The time period for the data collection was staggered with the senior nurses of the centers to avoid disruptions in their normal health delivery services. On each day of data collection, the rational of the study was clearly explained to participants. For each pregnant adolescent identified, a non-pregnant counterpart was available for the completion of the instrument at each center during the whole period. The completion of the instrument was done simultaneously in the conference rooms of these chosen health facilities. Standard instructions were read to all participants who agreed to provide information on the questionnaire. The trained research assistants and volunteered nurses helped with the administration of the questionnaire to some respondents, especially to those who needed assistance with the interpretation of some information in our local dialect due to their inadequate proficiency in English language. The data collection process commenced after briefing and inform consent agreement reached with the study participants and assurance that information obtained was solely for academic purpose. Participation was entirely voluntary and participants were asked to withdraw from the study at any time they felt uncomfortable. Other considerations such as anonymity, respect, and confidentiality were persevered at all stages of the data collection process by omitting any personal identifier from the questionnaire.

### Data Analysis

Each completed questionnaire was coded on pre-arranged coding sheet by the researchers to minimize errors. Data were cleaned and entered into SPSS version 21 for analysis. Bivariate analysis using chi-square test of independence was initially performed between the dependent variable (pregnancy status) and each of the independent variables (socio-cultural factors) to identify the independent variables that showed statistically significant association with the dependent variable. The independent variables that showed statistically significant association with the dependent variable were further put in a multiple regression model to determine their influence on the dependent variable. Odds ratio (OR) at 95% confidence intervals (CI) and *p*-values were obtained for significant variables in the bivariate analysis. All variables found to be significant at the bivariate level (*p* **≤** 0.05) were entered into a multivariate analysis model in order to control for the effect of confounding variables. For easy interpretation, all odds ratios <1 were reversed to get whole numbers and interpretations were done based on the reverse values.

## Results

### Socio-Demographic Characteristics of Respondents

The results of Table 1 show that with religion, majority of the pregnant and non-pregnant adolescents were Christians (95 and 94.5%), respectively. For educational level, most of the pregnant and non-pregnant adolescents had more than primary education (64 and 52%), respectively. With mothers' level of education, 61.2 and 85.7% of the pregnant and non-pregnant adolescents' mothers had more than primary education. In relation to the fathers' level of education, majority of pregnant adolescents fathers (70.1%) and the non-pregnant adolescents' fathers (89.3%) had more than primary education. Most of the pregnant and non-pregnant adolescents' mothers (77.5 and 88.4%) were employed. Similar results, 77.5% of the pregnant adolescents' fathers and 89.8% of non-pregnant adolescents' fathers were employed.

**Table 1:** Socio-demographic characteristics of respondents.

**Socio-demographic factors**	**Pregnant (*****n*** **=** **200)**		**Non-pregnant (*****n*** **=** **200)**	
	***N***	**%**	***N***	**%**
**RELIGION**				
Non-Christians	10	5	11	5.5
Christians	190	95	189	94.5
**EDUCATIONAL LEVEL**				
Primary education or less	5	2.5	2	1
>primary education	195	97.5	198	99
**MOTHER'S LEVEL OF EDUCATION**				
Primary education or less	59	38.8	24	14.3
>primary education	93	61.2	144	85.7
**FATHER'S LEVEL OF EDUCATION**				
Primary education or less	40	29.9	16	10.7
>primary education	94	70.1	133	89.3
**MOTHER'S EMPLOYMENT STATUS**				
Unemployed	43	22.5	23	11.6
Employed	148	77.5	176	88.4
**FATHER'S EMPLOYMENT STATUS**				
Unemployed	42	24.3	20	10.2
Employed	131	75.7	177	89.8

### Socio-Cultural Factors Associated With Adolescent Pregnancy

Table 2 shows the results of a bivariate analysis of the socio-cultural factors that influence pregnancy among adolescents in the KEEA Municipality. In the bivariate analysis, all the independent variables had statistically significant association with adolescent pregnancy. In relation to the level of strictness of rules and regulations in family, the highest proportion of pregnancy (79.2%) was recorded among adolescent girls whose families had no strict rules and regulations. Again, 56% of adolescent girls who do not have the freedom within the family to discuss issues related to sexuality were pregnant. Adolescent girls whose religions support sex before marriage recorded the highest proportion of pregnancy (64.7%). Above average (61.3%) of adolescent girls indicated that their peers influence them to engage in sexual intercourse, those whose parents would be happy if they got pregnant (86.7%) and those whose siblings will be happy if they got pregnant (87.1%) were those who were pregnant. From the chi-square analysis, level of strictness of rules and regulations in family [χ^2^_(1)_ = 62.010, *p* < 0.05], freedom within the family to discuss issues related to sexuality [χ^2^_(1)_ = 9.600, *p* < 0.05], religious support of sex before marriage [χ^2^_(1)_ = = 4.312, *p* < 0.05], peer influence to engage in sexual intercourse [χ^2^_(1)_ = 7.793, *p* < 0.05], reaction of parents toward pregnancy [χ^2^_(1)_ = 70.064, *p* < 0.05], and reaction of siblings toward pregnancy [χ^2^_(1)_ = 66.702, *p* < 0.05] showed statistically significant relationship with pregnancy status.

**Table 2:** Bivariate analysis of socio-cultural factors associated with adolescent pregnancy.

**Socio-cultural factors**	**Pregnant %**	**Non-pregnant %**	**Chi-square (***χ^2^***)**	***P*-Value (95% CI)**
*Level of strictness of rules and regulations in family*			62.010	0.000^*^
Not strict	79.2	20.8		
Strict	36.7	63.3		
*Freedom within the family to discuss issues related to sexuality*			9.600	0.002^*^
No	56	44		
Yes	40	60		
*Religious support of sex before marriage*			4.312	0.038^*^
No	46	54		
Yes	64.7	35.3		
*Peer influence to engage in sexual intercourse*			7.793	0.005^*^
No	45.7	54.3		
Yes	61.3	38.7		
*Reaction of parents toward pregnancy*			70.064	0.000^*^
Sad	38.1	61.9		
Happy	86.7	13.3		
*Reaction of siblings toward pregnancy*			66.702	0.000^*^
Sad	38.8	61.2		
Happy	87.1	12.9		

* Significant results

In the multivariate analysis, five independent variables (level of strictness of rules and regulations in family, freedom within the family to discuss issues related to sexuality, peer influence to engage in sexual intercourse, attitude of parents toward pregnancy, and attitude of siblings toward pregnancy) showed statistically significant influence on adolescent pregnancy. The full model containing all the predictors was statistically significant [χ2 _(6)_ = 156.5, *p* < 0.05], indicating that the model was able to distinguish between respondents who were pregnant and those who were not pregnant. The model as a whole explained between 35% (Cox & Snell *R*^2^) and 47% (Nagelkerke *R*^2^) of variance in pregnancy status (see Table 3). Comparison of the socio-cultural factors associated with pregnancy among the respondents shows that non-pregnant adolescents were 7 times more likely to belong to families with strict rules and regulations compared to pregnant adolescents [OR = 0.14, 95% CI = [0.07–0.25], *p* **≤** 0.01]. Additionally, non-pregnant adolescents were 3 times more likely to have freedom within the family to discuss issues related to sexuality compared to pregnant adolescents [OR = 0.33, 95% CI = [0.18–0.59], p **≤** 0.01]. Again, pregnant adolescents were 3 times more likely to be influenced by peers to engage in sexual intercourse [OR = 2.63, 95% CI = [1.46–4.74], *p* **≤** 0.001]. Similarly, pregnant adolescents were 3 times more likely to have parents with positive reaction toward adolescent pregnancy compared to non-pregnant adolescents [OR = 2.97, 95% CI = [1.15–7.70], *p* **≤** 0.05]. Further, pregnant adolescents were 8 times more likely to have siblings with positive reaction toward adolescent pregnancy [OR = 7.74, 95% CI = [2.59–21.4], *p* **≤** 0.001] compared with non-pregnant counterparts.

**Table 3:** Multivariate analysis of socio-cultural factors associated with adolescent pregnancy.

	**Pregnant %**	**Non-pregnant %**	**B**	**Wald**	**OR (CI)**	***p*-value**
Pseudo *R*^2^	0.35–0.47					
χ2	156.5					
*p*-value	0.000					
**LEVEL OF STRICTNESS OF RULES AND REGULATIONS IN FAMILY**						
Not strict	79.2	20.8	−1.991	41.407	Ref	
Strict	36.7	63.3			0.14 (0.07–0.25)	0.000^*^
**FREEDOM WITHIN THE FAMILY TO DISCUSS ISSUES RELATED TO SEXUALITY**						
No	56	44	−1.120	13.850	Ref	
Yes	40	60			0.33 (0.18–0.59)	0.000^*^
**RELIGIOUS SUPPORT OF SEX BEFORE MARRIAGE**						
No	46	54	0.719	2.551	Ref	
Yes	64.7	35.3			2.05 (0.85–4.96)	0.110
**PEER INFLUENCE TO ENGAGE IN SEXUAL INTERCOURSE**						
No	45.7	54.3	0.965	10.272	Ref	
Yes	61.3	38.7			2.63 (1.46–4.74)	0.001^*^
**REACTION OF PARENTS TOWARD PREGNANCY**						
Sad	38.1	61.9	1.089	5.019	Ref	
Happy	86.7	13.3			2.97 (1.15–7.70)	0.025^*^
**REACTION OF SIBLINGS TOWARD PREGNANCY**						
Sad	38.8	61.2	2.007	13.875	Ref	
Happy	87.1	12.9			7.44 (2.59–21.4)	0.000^*^

* Significant results

## Discussion

This study sought to identify and examine what socio-cultural factors influence pregnancy among selected adolescent girls in one of the endemic areas (KEEA Municipality) of the Central Region of Ghana. Findings show that non-pregnant adolescents are more likely to belong to families with strict rules and regulations. We do infer that adolescents whose parents or guardians have well-laid down monitoring and supervision guide (i.e., through rules and regulations) for their children may reduce adolescents' pregnancy risk such that these girls may refrain from sexual engagement (abstinence), postpone sexual intercourse (i.e., later sexual debut) and/ or have no sexual partners (29, 30). This finding corroborates other studies [e.g., (18, 20, 31–33)] that reported inverse relationship between parental monitoring (i.e., strict rules and regulations) and adolescent pregnancy risk (i.e., adolescents not having sexual intercourse or having sex less often. However, few studies have reported contradictory findings by failing to show the association between parental strict control and adolescent pregnancy risk. For example, Resnick et al. (34) reported that parents' presence at home before and after school, at dinner, and at bedtime (as regulatory mechanism) was not related to adolescents' pregnancy history. Likewise, mothers' strictness and rules were not related to daughters' sexual intercourse status, after other variables were controlled (35). A plausible reason for these mixed findings is that parental control is connected with negative adolescent behavior, including sex if it is excessive or coercive (29). Evidence supports the linkage between adolescent pregnancy risk with parents' intrusive psychological control. For instance, Miller et al. (33) found that adolescents who perceived their parents to be “very strict” with “many rules” were more likely to have had sexual intercourse than their peers who perceived their parents to be more flexible or exhibit moderate parental control. More research is thus required for additional conceptual and empirical clarification.

Consistent with previous studies [e.g., (30, 35, 36)], the freedom within the family to discuss issues related to sexuality was related to adolescent pregnancy in this study. Specifically, non-pregnant adolescents were more likely than pregnant adolescents to have the freedom within the family to discuss issues related to sexuality. There is enough evidence to suggest that an open, positive, and frequent parent/child communication about sexuality is connected with adolescents not having sexual intercourse, postponing their sexual debut, and/ or having fewer sexual partners will reduced risk of pregnancy. Comparatively, there are some studies that have reported conflicting findings; that is, the amount or frequency of parent/child sexual communication being associated with sons (37, 38) or daughters (39) or both (40) being more likely to have sexual intercourse, thus increasing pregnancy risk among adolescents. Methodological limitations (e.g., different measures [i.e., ever talked about sex (communication content), regularity of sexual communication, number of topics discussed as well as temporal ordering]) have been cited as potential reasons accounting for the complexity surrounding parent/child communication and adolescent pregnancy risk inconclusive findings (29, 30, 41). Future research should consider the potential interactive effects between these aforementioned measures toward the prediction of adolescents' pregnancy risk using longitudinal designs compared to cross-sectional studies that currently dominate literature.

Our current study also demonstrated that peer pressure to engage in sexual intercourse was found to be a risk factor for adolescent pregnancy. Previous studies have likewise recounted that peer influence can support in the promotion or prevention of adolescent pregnancy through multiple pathways (e.g., attitudes and behaviors; perceptions) and that perceptions of their peers' attitudes and behaviors were found to be more influential toward increased pregnancy risk (22, 42–44). Hayes suggested that if an adolescent has confidence in his or her peers as sexually active although they might not be, this belief will increase the adolescent's prospect of engaging in sexual intercourse. This premise is consistent with our finding where adolescent pregnancy was ascribed to peer influence to engage in sexual intercourse by 61% of our study participants. Similarly, having a boyfriend, especially if adolescent girls are dating an older male counterpart has been associated with an increased risk of adolescent pregnancy (43).

Positive attitude of parents and siblings toward pregnancy was also found to be a risk factor for adolescent pregnancy. Some researchers have argued cogently that parents' sexual attitudes and values determine whether these young girls would have sexual intercourse, the onset of their sexual debut, the number of sexual partners encountered, their use of contraceptives, and whether they have ever been pregnant [e.g., (18, 22, 45–48)]. For instance, Dittuset et al. (49) highlighted that sexual intercourse was deferred among Black adolescents when their fathers were perceived as condemning any sort of sexual engagement. Reasonably, parents' positive attitudes and values for their adolescent children to avoid pregnancy (i.e., sexual abstinence) are efficiently transferred when parents have a close bond (i.e., connectedness) with their children (48, 50). Similarly, the extent to which younger siblings exhibit risky sexual behaviors, manifest strongly if older siblings have had sexual intercourse and have experienced an adolescent pregnancy or birth in their family history (31, 51–53). Again, sibling modeling effects on adolescents' sexual attitudes and sexual behavior are most prevalent when siblings interact frequently and have a warm and amiable relationship (18). It is therefore interesting to find that pregnant adolescents are more likely to have siblings who support adolescent pregnancy compared to non-pregnant adolescents. Thus, within a family where parents and siblings are happy when an adolescent girl gets pregnant, may influence the sexual behavior of adolescents in the family.

## Limitations

This study may be subject to some limitations. The study was restricted to five health facilities in the KEEA Municipality of the Central Region of Ghana. Therefore, the findings cannot be generalized to other parts or areas of this Municipality or the country. By using a case-control design, temporal relationship between cause and effect cannot be determined from the outcomes (54). Additionally, this approach may be beset with both sampling errors and recall bias (55). Further, by assessing predictor variables retrospectively, there is the likelihood of a biased assessment of their presence and significance by participants or the investigator or both. These challenges were addressed by sampling cases and controls that have similar characteristics aside pregnancy status. Again, respondents were given adequate time to recall various events that had occurred in the past.

### Practical Implications

Some of the contextual indicators (e.g., parental supervision [i.e., adopting strict rules and regulations]), parental communication, peer influence, parental and siblings' attitudes do make a difference toward risk of adolescent pregnancy. However, there are other multifaceted variables (e.g., family and neighborhood connectedness) that increase pregnancy risk, other adolescent sexual and reproductive health concerns seem to be complicated and inconclusive (56). For example, diverse family, societal, cultural, and religious influences create unfriendly environment for the discussion of adolescent sexuality related issues. Many societies (e.g., Ghana) across the Sub-Saharan region hold an entrenched position of disapproval toward adolescent sexual activity. This is often shown through stigmatization and stereotypical behaviors which increase the risk of adolescent mothers not accessing health and other social services in the communities, thus become more socially isolated and rejected. Based on our findings, we believe that reducing adolescent pregnancy rates warrant comprehensive multifaceted strategies. Attention should be drawn to the Municipal Assembly, educational and health directorates within KEEA toward improving sex education and access to sexual and reproductive health services. Attempts should also target addressing economic inequalities, improving education and employment opportunities for the large adolescent population in KEEA Municipality and perhaps other deprived areas in the country. Programs such as specific competency-based skill based education and training should be implemented. Such programs should include academic and social skills, particularly those that would include adolescents' parents, family and the broader community.

## Conclusion

Findings from the study show that adolescent girls in the KEEA Municipality are likely to be exposed to the risk of pregnancy with the existence of negative socio-cultural norms. Most of the pregnant adolescents had no freedom within the family to discuss issues related to sexuality, were influenced by peer influence to engage in sexual intercourse and had parents and siblings with positive attitudes toward adolescent pregnancy compared to non-pregnant adolescents. Specific strategies need to involve multifaceted interventions that include education, competency-based skill training and support for young people, especially pregnant adolescents. Additionally, existing ethics and positive cultural values at the community level should be enforced. Further research is required to determine which other factors would help us better understand circumstances that may lead to adolescent pregnancies in other areas of the region and perhaps among other subgroups.

## Ethics Statement

Ethical approval for this study was approved by the Research and Ethics Committee of University of Cape Coast, Ghana (UCCIRB/CES/2016/04) and the Ghana Health Service Ethics Review Committee (GHS-ERC: 13/12/2016).

## Author Contributions

BA conceived the study. BA and FS designed and performed the analysis and the write up on data and methods. BA, JH, JM, A-AS, and FS designed the first draft of the manuscript. BA, JH, JM, A-AS, FS, TS, and TH revised and proof read the manuscript for intellectual content and gave consent for the final version to be published.

### Conflict of Interest Statement

The authors declare that the research was conducted in the absence of any commercial or financial relationships that could be construed as a potential conflict of interest.
